# Mechanisms of bacterial inhibition and tolerance around cold atmospheric plasma

**DOI:** 10.1007/s00253-023-12618-w

**Published:** 2023-07-08

**Authors:** Hao Zhang, Chengxi Zhang, Qi Han

**Affiliations:** 1 State Key Laboratory of Oral Diseases & National Clinical Research Center for Oral Disease, West China Hospital of Stomatology, Sichuan University, Chengdu, 610041 People’s Republic of China; 2 Department of Oral Pathology, State Key Laboratory of Oral Diseases and National Clinical Research Center for Oral Diseases, West China Hospital of Stomatology, Sichuan University, Chengdu, 610041 People’s Republic of China

**Keywords:** Cold atmospheric plasma, Bacterial tolerance, Bacterial resistance, Biofilm, Bactericidal application

## Abstract

**Abstract:**

The grim situation of bacterial infection has undoubtedly become a major threat to human health. In the context of frequent use of antibiotics, a new bactericidal method is urgently needed to fight against drug-resistant bacteria caused by non-standard use of antibiotics. Cold atmospheric plasma (CAP) is composed of a variety of bactericidal species, which has excellent bactericidal effect on microbes. However, the mechanism of interaction between CAP and bacteria is not completely clear. In this paper, we summarize the mechanisms of bacterial killing by CAP in a systematic manner, discuss the responses of bacteria to CAP treatment that are considered to be related to tolerance and their underlying mechanisms, review the recent advances in bactericidal applications of CAP finally. This review indicates that CAP inhibition and tolerance of survival bacteria are a set of closely related mechanisms and suggests that there might be other mechanisms of tolerance to survival bacteria that had not been discovered yet. In conclusion, this review shows that CAP has complex and diverse bactericidal mechanisms, and has excellent bactericidal effect on bacteria at appropriate doses.

**Key points:**

*• The bactericidal mechanism of CAP is complex and diverse.*

*• There are few resistant bacteria but tolerant bacteria during CAP treatment.*

*• There is excellent germicidal effect when CAP in combination with other disinfectants.*

## Introduction

Bacterial infection is a difficult problem in medical treatment and can be life-threatening in severe cases (Holland et al. 2014; Nasr et al. 2013). Antibiotics are considered to be one of the most important means of treating bacterial infections (Khardori et al. 2020a, b). With the frequent use of antibiotics on a global scale, unregulated antibiotic use is inevitable. The misuse of antibiotics was once widespread, making bacterial resistance to antibiotics one of the most significant threats to human life and health (Huemer et al. 2020). A possible solution is to find a new means of bactericidal that is independent of bacterial resistance to antibiotics. Cold atmospheric plasma (CAP) has been found to have this potential (Gilmore et al. 2018).

Plasma is considered to be the fourth state of matter after solid, liquid, and gaseous states, which consists of a mixture of excited molecules, charged particles, reactive oxygen species (ROS), reactive nitrogen species (RNS), and ultraviolet (UV) (Graves 2014; Mai-Prochnow et al. 2014). Based on the difference in the relative energy levels, it can be divided into thermal plasma and non-thermal plasma. Due to high temperature and high energy density, thermal plasma is very destructive and can be used to degrade various types of waste, while non-thermal plasma, which is also called CAP, has better prospects in clinical medicine and food safety because of their lower temperature characteristics (Bernhardt et al. 2019; Braný et al. 2020).

Currently, many studies focused on application of CAP in the field of bactericidal; however, the mechanism of the interaction between CAP and bacteria is still a perplexing issue (Zhu et al. 2020). In this paper, we summarize the mechanisms of bacterial killing by CAP in a systematic manner, and discuss the responses of bacteria to CAP treatment that are considered to be related to tolerance and their underlying mechanisms, review the recent advances in bactericidal applications of CAP, filling the research gap in this field to a certain extent.

## Mechanism of destruction of bacterial components by CAP

Several CAP inhibition mechanisms have been described based on the classification of cellular structural components, including oxidation and perforation of cell membrane, degradation and modification of proteins, modification and chain-breaking of nucleic acid molecules, and disruption of extracellular polymeric substances (EPS) and interference with quorum sensing (QS) on biofilms (Krewing et al. 2019a; Mai-Prochnow et al. 2015). In this subsection, we will systematically discuss the mechanisms of CAP damage to different bacterial components.

### Mechanism of CAP action on cell membrane/cell wall

The most common mechanism by which CAP affects cell membranes is by oxidizing the lipid components of the cell membrane. Membrane lipids, located on the outer side of the cell membrane, are considered to be the most susceptible macromolecules in the cell to physical stress (Mai-Prochnow et al. 2016). ROS and RNS in CAP were found to induce membrane lipid oxidation to malondialdehyde (MDA). Interestingly, MDA is also a highly reactive substance that can react with intracellular components. At the same time, compared with most of the CAP-produced reactive species, MDA have relatively higher stability, which facilitates its diffusion from the site of production into the cell interior and reacts with intracellular proteins and DNA (Alkawareek et al. 2014b; Joshi et al. 2011). Therefore, MDA can be regarded as a secondary toxic messenger of the CAP-generated reactive species, which is produced by CAP oxidation of membrane lipids and diffuses into the cell interior to react with proteins and DNA, thereby amplifying CAP damaging effects inside the cell (Alkawareek et al. 2014b).

In addition to oxidizing lipids in cell membranes, CAP can also cause direct perforation damage to cell membranes through the etching effect. The so-called etching effect refers to the bombardment of charge ions or discharge gases during CAP generation, which can lead to the breaking of chemical bonds of compounds, especially hydrocarbons, and the formation of holes and perforations in the cell membrane. The mechanism by which the etching effect leads to perforation of the cell membrane is considered to be related to the accumulation of charge on the cell membrane, which is considered to be dominated by the charged particles with the electric field in the CAP. When the electrostatic stress generated by the accumulated charge on the cell membrane exceeds the tensile strength of the cell membrane, bacteria are inactivated by the rupture of the cell membrane (Mendis et al. 2000; Smolkova et al. 2018).

When CAP acts separately with Gram-positive and Gram-negative bacteria, the inhibition effect of CAP on Gram-negative bacteria is usually better than that on Gram-positive bacteria. This is attributed to the fact that the two bacteria have distinct differences in cell wall structure, which allows CAP to have different bactericidal mechanisms. Gram-negative bacteria possess an outer membrane and a thinner peptidoglycan layer. The outer membrane components such as LPS and proteins are sensitive to ROS; therefore, CAP can cause disruption of outer membrane and then lead to the destruction of cell wall and cell membrane. In contrast, Gram-positive bacteria do not contain an outer membrane and are enveloped by a thicker layer of peptidoglycan, which is less susceptible to oxidative damage. Therefore, the cell wall of Gram-positive bacteria is not easily damaged by CAP directly, but the active molecules generated can enter the cell through the interstitial spaces into the cell interior, causing oxidative damage to intracellular molecules (Huang et al. 2020; Kang et al. 2021). In summary, CAP causes bacterial damage to Gram-negative bacteria mainly by damaging the cell membrane and inducing leakage of intracellular material, while for Gram-positive bacteria, CAP enters the cell interior directly and causes oxidative damage to intracellular material resulting in bacterial death.

### Mechanism of CAP action on proteins

CAP damage to proteins may require the accumulation of several events, such as amino acid modifications or disruption of hydrogen bonds in the tertiary structure of the protein, to severely alter the normal structure of the protein, thus stopping the normal function of the protein (Alkawareek et al. 2014b). Recent studies have found that amino acid modification plays a major role in CAP-induced protein damage. The modification of amino acids by CAP has been attributed to the oxidative modification by ROS and RNS and the modified amino acids mainly exhibit hydroxylation, carbonylation, and nitration (Dezest et al. 2017; Persson et al. 2020; Zhou et al. 2016). In a review of Zhou et al., the oxidative modification of amino acids by plasma is discussed in detail (2016). Modification of amino acids can lead to changes in the secondary structure of proteins. Protein secondary structure mainly includes α-helix, β-folding, β-turning, and random coiling, and the CAP-treated protein secondary structure exhibits changes in the decrease of the specific weight of α-helix and the increase of the specific weight of random coiling (Qian et al. 2022; Soler-Arango et al. 2019; Tolouie et al. 2018). It is worth mentioning that the changes in protein secondary structure are considered to be mainly attributed to long-lived substances in the CAP, such as hydrogen peroxide and ozone (Qian et al. 2022). Also, amino acid modifications promote the formation of protein-protein cross-links and DNA-Protein crosslinks (DPCs). The formation of protein-protein cross-links wrapping a small number of active sites reduces enzymatic activity (Zhang et al. 2015); and the interference of DPCs with DNA replication and transcription on DNA repair mechanisms is particularly challenging and highly lethal to cells (Guo et al. 2018).

### Mechanism of CAP action on nucleic acid molecules

CAP damage to nucleic acid molecules can be divided into direct damage and indirect damage effects. Direct damage refers to the direct reaction of CAP active components with nucleic acid molecules, mainly referring to nucleotide chain breakage; indirect damage refers to the damage to nucleic acid molecules caused by the reaction products of CAP with other molecules.

CAP -induced nucleotide chain breakage is mainly attributed to ROS and RNS. ROS and RNS can directly attack the deoxyribose backbone, extract H atoms from deoxyribose and form deoxyribose radicals, and further break the N-glycosidic bonds leading to the release of nitrogenous bases, eventually leading to the breakage of the deoxyribonucleotide chain (Arjunan et al. 2015). Nucleotide breaks include single-strand breaks (SSB) and double-strand breaks (DSB). ROS and RNS generally do not induce DSB directly, but rather SSB, which is reversible and easily fixed by cellular repair enzymes; whereas SSB can be converted into DSB during the normal replication of DNA, and DSB is more difficult for cells to repair effectively and may therefore be lethal to viable cell (Alkawareek et al. 2014a; Arjunan et al. 2015). In addition to ROS and RNS, UV photons in the CAP component have damaging effects on nucleic acid molecules. UV photons can induce the formation of thymine dimers in DNA chains, which leads to structural abnormalities in bacterial nucleic acid molecules and can effectively inhibit the replication ability of bacteria (Salgado et al. 2021).

By attacking lipids or proteins, CAP is also able to have an indirect damaging effect on nucleic acid molecules. As mentioned above, the oxidation product MDA can react with DNA, leading to DNA damage and eventually making the cell dead (Ranjbar et al. 2020), while modification of amino acids contributes to the formation of DPCs, which have significant interference with DNA replication and transcription (Guo et al. 2018). ROS and RNS in CAP are also known to damage DNA replicase and DNA polymerase, thus slowing down the process of DNA replication and repair (Arjunan et al. 2015).

### Mechanism of CAP action on biofilm

Biofilms are complex communities of microbial cells that are attached to a tissue matrix composed primarily of EPS and can capture or spontaneously produce EPS, including polysaccharides, proteins, eDNA, and phospholipids (Gilmore et al. 2018). Most bacteria grow as biofilms rather than as planktonic cells because biofilms offer the advantages of community life and therefore can better survival in harsh environments (Mai-Prochnow et al. 2021). Similar to planktonic bacteria, bacteria in biofilms are subject to the action of CAP, leading to the destruction of cell membranes, proteins, and nucleic acid molecules (Zhu et al. 2020). However, in addition to these, CAP has additional mechanisms of bacterial inhibition on biofilms, specifically the disruption of EPS and interference with QS.

**Fig. 1:**
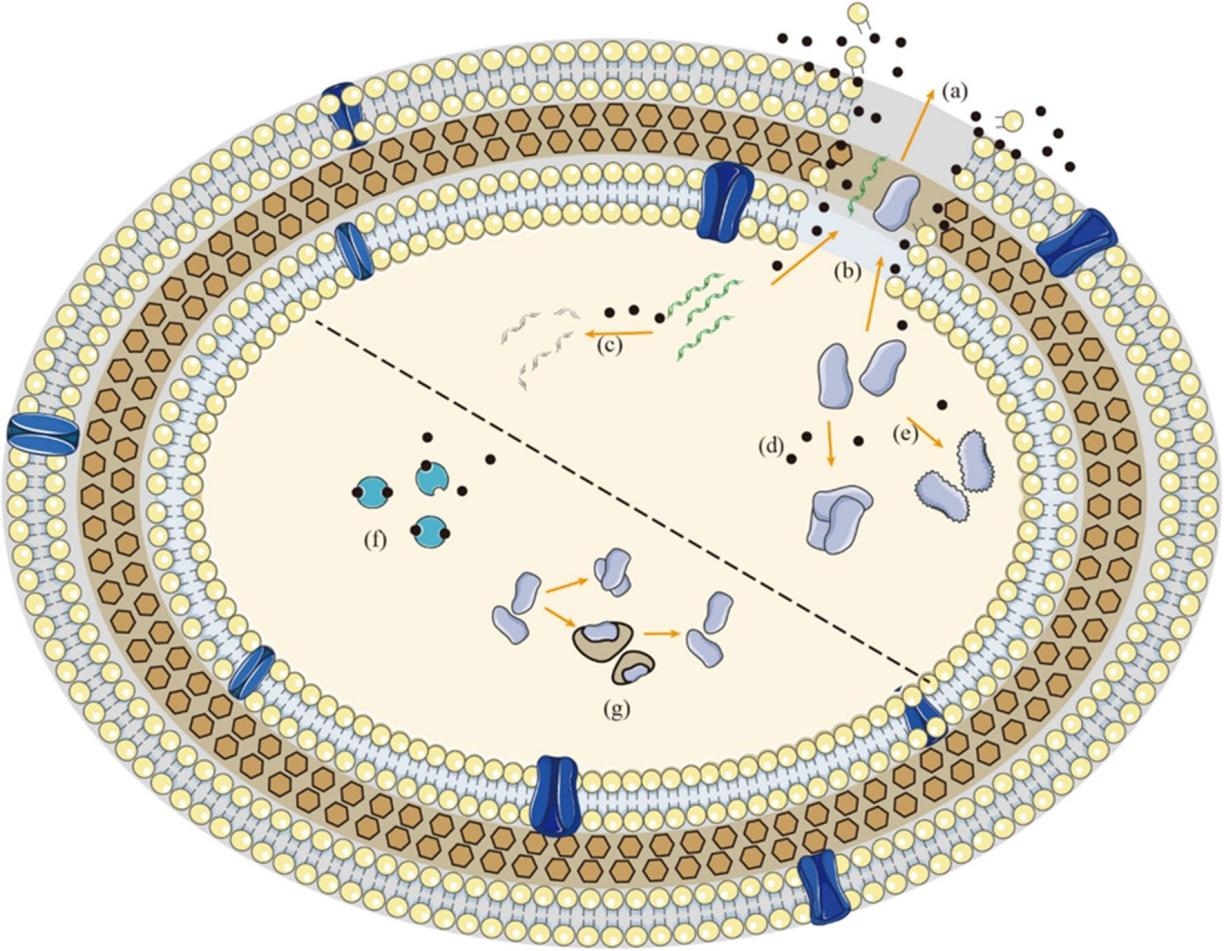
Mechanism of action between CAP and planktonic bacteria. **a** Perforation of cell membrane induced by ROS and RNS. **b** Leakage of proteins and DNA in the cell through the pores. **c** Breakage of nucleotide chains caused by oxidative stress. **d** Modifications of proteins promote the formation of protein–protein cross-links. **e** Destruction of proteins structure induced by CAP. **f** Bacteria fight against ROS and RNS mainly through antioxidant enzymes. **g** Bacteria prevent the formation of protein–protein cross-links through head shock proteins. Black spots represent ROS and RNS in the CAP

EPS encapsulated in biofilms can limit the mobility of biofilm cells and bring them into close contact, thus creating conditions for strong cell-cell interactions to occur, including cell-cell communication, intercellular horizontal gene transfer, formation of synergistic micro consortia and so on (Mai-Prochnow et al. 2021). It can be said that EPS is the material basis for biofilm formation and function. Similar to bacterial components, EPS is also easily disrupted by the action of CAP. During CAP treatment, EPS will undergo peroxidative chain reactions of lipids, modification and degradation of proteins, and chemical bond breakage of carbohydrates, ultimately leading to the destruction of EPS (Khan et al. 2016; Khosravi et al. 2021). This biochemical alteration of EPS is mainly attributed to the oxidation of ROS and RNS. By disrupting the EPS, the adhesion of the biofilm to its anchoring surface is reduced, which in turn leads to the disruption or even disintegration of the three-dimensional structure of the biofilm (Soler-Arango et al. 2019). This indicates that CAP can transform bacteria from biofilm form into planktonic bacteria form, suggesting that CAP has the potential of bactericidal application in combination with other disinfectants, which has poor effect on biofilm but excellent effect on planktonic bacteria (Fig. 1).

The QS is a population-dependent intercellular signaling system in which intercellular communication is regulated by a small molecule called autoinducers, which can be viewed as a signaling molecule. In this system, as the number of bacterial cells increases, the accumulation of bacterial signaling molecules increases, allowing microorganisms to respond in a coordinated manner to the external environment and to regulate population behavior, including biofilm formation, production of virulence factors, resistance to disinfectant, etc. (Whiteley et al. 2017). The signaling pathway for QS consists of three components, the gene encoding the signaling molecule, the signaling molecule itself, and the signaling molecule receptor (Flynn et al. 2016). In recent studies, CAP was found to affect the synthesis of the signaling molecule and to disrupt the signaling molecule itself, thus interfering with QS. In an experiment by Cui et al., the expression of QS-related genes was found to be differentially down-regulated in bacteria after CAP treatment, suggesting that CAP can interfere with the synthesis of QS signaling molecules (2021); and in an experiment by Flynn et al., it was demonstrated that AHL molecules, one of the most typical autoinducers, can be time-dependent degradation and conversion to a series of by-products under CAP treatment, suggesting that CAP has a role in disrupting the signaling molecule itself (2016). By affecting the synthesis of the signaling molecule or by disrupting the signaling molecule itself, CAP can interfere with the QS and thus inhibit the production of virulence factors mediated by the QS, thus weakening the toxicity of biofilms and having a positive effect on the control of biofilm infections.

## Bacteriostatic mechanism of each component of CAP

CAP produces various components that can have bactericidal efficacy, including ROS, RNS, UV, temperature, pH, charged particles, and electric fields. Among these bactericidal factors, ROS and RNS are considered to mainly mediate the bactericidal effect. In this section, we will discuss in turn the mechanisms of action of CAP components in the process of bactericidal inhibition.

### Mechanism of action of ROS and RNS

ROS and RNS are highly reactive substances containing oxygen or nitrogen (Arjunan et al. 2015). ROS and RNS can be classified as long-lived substances or short-lived substances according to their duration of survival. Long-lived substances have a more stable chemical state but are less able to inactivate bacteria, while short-lived substances have a more active chemical state and are correspondingly more able to inactivate bacteria (Kondeti et al. 2018). Among ROS, hydroxyl radicals (·OH) have the highest redox potential, are the most reactive, and are considered to play an important role in CAP bacterial treatment (Arjunan et al. 2015), while among RNS, peroxynitrite (ONOO^−^) is representative of the highly reactive species, and in some views, CAP bactericidal action is considered to be directly related to peroxynitrite concentration (Zhou et al. 2018). Short-lived substances represented by hydroxyl radicals and peroxynitrite are closely related to long-lived substances, which are converted to long-lived substances after energizing reaction, and long-lived substances can also be converted to highly reactive short-lived substances after energy absorption. Thus, the ROS and RNS in CAP are in a complex state of reaction. A detailed review of the reactions between reactive species in CAP can be found in Mai-Prochnow et al. (2021). The complex reaction state between ROS and RNS also suggests that the inhibition of CAP cannot be attributed to one reactive substance alone, but is the result of the combined action of all reactive substance.

The bactericidal effect of ROS and RNS is attributed to their high reactivity and their ability to react sufficiently with polysaccharide, lipid, protein, and nucleic acid molecules in planktonic bacteria and biofilms to damage bacterial structures and thus cause damage, which has been discussed in more detail in the previous section. The high reactivity of ROS and RNS and their high dose concentrations in the CAP have led researchers to generally agree that they are the main active substances for the CAP to exert its bacterial inhibitory effects (Julák et al. 2018; Machala and Pavlovich 2018).

### Mechanism of action of UV on bacteria

UV is invisible light with a higher frequency than blue-violet light and can be classified as UVA (320–400 nm), UVB (280–320 nm), and UVC (< 280 nm) depending on the wavelength. In general, the shorter wavelength UVC photons show higher energy and have stronger bactericidal effect, while UVA and UVB are relatively less effective (Motyka et al. 2018). UVC photons are considered to be an effective bactericidal substance because of their ability to induce the formation of thymine dimers in DNA strands, thereby interrupting DNA replication (Salgado et al. 2021). In addition, UV photons are also suggested to disrupt the protein content of outer membranes, causing the breaking of chemical bonds in the outer cellular structures (Chen et al. 2020; Costello et al. 2021).

Although UV light can be detected in the compositional analysis of CAP, scholars mostly agree that UV light is not the main substance that contributes to bacterial inactivation. On the one hand, the concentration of UV photons in the CAP is low and significantly lower than the effective dose of UV that can effectively kill bacteria (Estifaee et al. 2019). On the other hand, the CAP-generated UV photons are shown to consist not of short-wave UV-C but of long-wave UV-A and UV-B and their antimicrobial efficiency is significantly lower than that of UV-C (Hertwig et al. 2017; Sakudo et al. 2017). In the experiments of Wang et al., treating bacteria with UV light isolated from the CAP alone for 30 min resulted in a reduction of only 1.47 log, whereas bacteria could be reduced by the CAP by more than 6 log for the same amount of time (2018).

Although UVC photons are not the main active substance in CAP, UVC has an indirect role in the CAP germicidal process, acting in concert with other germicidal molecules. UVC is considered to promote the formation of ROS, in particular by inducing the decomposition of hydrogen peroxide (H_2_O_2_) to produce hydroxyl radicals. Under UV irradiation, hydrogen peroxide can absorb large amounts of UV energy at short wavelengths and subsequently decompose into highly reactive hydroxyl radicals (Nicol et al. 2020). In addition, UV light is also considered to affect the EPS of biofilms, destroying or degrading the upper layer of the biofilm matrix and aiding the penetration of ROS and RNS into the deeper structures of the biofilm (Soler-Arango et al. 2019).

### Mechanism of action of temperature on bacteria

There is some heating during CAP treating, but the temperature fluctuations are small. Especially when in clinical treatment, it is necessary to control the temperature of CAP near the body surface temperature to avoid the side effect of high temperature; in this case, the influence of temperature on bacteria is very small (Seo et al. 2017). It is known that according to the pasteurization method, achieving standard sterilization conditions requires continuous treatment at 60 °C for 30 min, whereas for CAP inhibition, satisfactory inhibition is usually achieved in 3–5 min, and is therefore unsatisfactory both in terms of temperature and treatment time (Boekema et al. 2021). Therefore, although the increase in temperature is believed to contribute to the generation of ROS (Kawano et al. 2018), it is generally accepted that the CAP-induced temperature increase is not the main reason for CAP-driven bacterial inactivation.

### Mechanism of action of pH on bacteria

It has been reported that the pH of the bacterial surroundings fluctuates after CAP treatment and mostly tends to acidify. CAP-induced acidification is often considered to be related to nitrogen oxides in the CAP gas phase, which can dissolve in water forming nitrate ions and nitrite ions, and these compounds form nitrate and nitrite, leading to a reduction in pH (Olszewski et al. 2014). In addition to nitrogen oxides, hydroxyl radicals are also considered to be involved in the acidification of CAP (Royintarat et al. 2020). Nevertheless, in a small number of experiments, CAP was found to have an alkalization effect on the treated solution (Kondeti et al. 2018; Motyka et al. 2018). A common denominator of these studies that found an increase in solution pH was that the solutions being treated by CAP were all sodium chloride (NaCl) solutions. The alkalization of solutions is suggested to be related to the electrolysis of highly electrolytic solutions, where the production of hydroxide radical ions (OH^−^) in the electrolysis reaction increases the pH of the solution.

It is worth noting that although extreme pH conditions can induce DNA double-strand breaks, which subsequently cause bacterial death (Arjunan et al. 2015), no significant killing effect of pH changes on bacteria was observed when the bacteria were incubated alone in the post-CAP-induced pH environment (Kondeti et al. 2018; Wang et al. 2019). It is due in large part to the fact that the pH does not reach the inactivation range under CAP induction and the CAP treatment time was usually within 3–5 min, which was too short to provide sufficient time for adequate bacterial inactivation. However, CAP-induced changes in pH environment have a facilitative effect on CAP-mediated bactericidal activity, especially under acidic conditions. Under low pH conditions, cell membrane permeability increase and reactive molecules are considered to penetrate cell walls more readily (Li et al. 2017; Royintarat et al. 2020). At the same time, the reactivity of some active molecules is highly dependent on pH, such as peroxynitrite which requires hydrogen ion-catalyzed decomposition to produce free radicals for its bactericidal action (Lukes et al. 2014). These studies suggest that CAP-induced pH condition has a certain auxiliary effect on bactericidal.

### Electric fields and the mechanism of action of charged particles on bacteria

The bactericidal mechanism of CAP is also considered to involve high electric fields and discharged particles (Banaschik et al. 2016; Modic et al. 2017), and the formation of high electric fields and discharged particles are considered to be closely linked to their bactericidal effects. Charged and neutral particles are formed during CAP generation, and these particles induce a strong local electric field during propagation (Dezest et al. 2017). Both charged particles and electric fields are considered to be able to disrupt cell membranes due to their electrostatic effect. Charged particles on the cell surface can generate electrostatic stresses that can cause structural changes in the cell membrane. When the electrostatic stresses exceed the tensile strength of the cell membrane, it can lead to perforation of the cell membrane (Motyka-Pomagruk et al. 2021). Similarly, electric fields can induce transmembrane potentials across the cell membrane, which can induce electroporation when the transmembrane potential exceeds a critical value (Dezest et al. 2017; Estifaee et al. 2019). When sterilization is carried out by an electric field alone, the induced cell membrane rupture tends to occur at the cell poles because of the higher electric field density at the cell poles (Banaschik et al. 2016), in contrast to CAP-induced cell membrane rupture which is randomly distributed across the cell, indirectly suggesting that CAP-induced cell membrane rupture is not entirely caused by the electric field. In summary, although electric fields and charged particles are considered to be able to induce bacterial death, this bactericidal effect is not efficient and does not completely explain the CAP-induced bactericidal effect.

## The causes of survival bacteria formation in CAP

Because of the complex and diverse bactericidal mechanisms of CAP, which can act on multiple targets when treating bacteria, CAP is considered to be less susceptible to the development of drug resistance (Modic et al. 2017; Nicol et al. 2020). However, survival bacteria can still occur after CAP treatment, especially when treating biofilms (Paldrychová et al. 2020). The emergence of survival bacteria depends mainly on the instrumentation parameters of the CAP, the bacteria themselves, and the surrounding environment in which the bacteria are located (Wu et al. 2020; Zhu et al. 2020). The instrumentation parameters and the treatment environment can influence the concentration dose of CAP when interacting with bacteria, and at low doses of CAP, there is a tendency for survival bacteria to emerge. In this section, however, we will systematically discuss the mechanisms of tolerance to CAP by the bacteria, from the perspective of the bacteria themselves.

### Anti-oxidative stress

Ancient microbes survived on Earth in anoxic form, and as atmospheric oxygen levels gradually increased, microbes underwent a series of long-term adaptations, eventually evolving an antioxidant system, which has been inherited into contemporary microbes (Khademian and Imlay 2021). In addition to the threat of oxygen from the atmosphere, the threat of oxygen from within bacteria cannot be ignored, a typical example being the production of ROS during respiration, hence the need for bacteria to have a strong antioxidant defense system (Shimizu and Matsuoka 2019). The antioxidant pathways of bacteria can be divided into enzymatic systems and non-enzymatic. The enzymatic system consists of various antioxidant enzymes, mainly catalase, peroxidase, superoxide dismutase, ascorbate peroxidase, etc.; the non-enzymatic system consists mainly of various antioxidants that can directly reduce ROS, such as pigments, phenols, ascorbic acid, and glutathione (Kashef and Hamblin 2017).

Intracellular antioxidant enzymes play an important role in the reactions involved in the reduction of ROS. In the intracellular antioxidant enzymes mainly include catalase and superoxide dismutase. The main role of catalase is to degrade hydrogen peroxide and convert it to water and oxygen (Van Acker and Coenye 2017). Superoxide dismutase is another important antioxidant enzyme. Under oxidative stress superoxide dismutase converts superoxide to hydrogen peroxide which is subsequently further degraded by catalase (Kashef and Hamblin 2017). Analysis of gene expression in CAP-treated survival bacteria revealed that genes associated with catalase production were significantly upregulated (Liao et al. 2020; Vaze et al. 2017), while genes which is responsible for superoxide dismutase production, was also upregulated, but to a relatively insignificant extent (Lee et al. 2019; Motyka et al. 2018). The comparison of gene expression results may suggest that catalase plays a more important role than superoxide dismutase in the formation of survival bacteria. Catalase is suggested to play an important role in the formation of survival bacteria, not only because hydrogen peroxide is a long-lived substance that is abundant in the CAP but also because catalase has a reducing effect on other ROS and RNS in addition to degrading hydrogen peroxide (Alshraiedeh et al. 2016). In contrast, superoxide dismutase contributes less to the formation of survival bacteria, which may be partly due to the fact that the superoxide anion is less permeable compared to hydrogen peroxide; in addition, it may also be due to the fact that superoxide is more active and has often been converted directly into other substances during transport, resulting in a lower actual effect of superoxide on the cell (Vaze et al. 2017). In addition to catalase and superoxide dismutase, other reductases which also play a role in the tolerance to oxidative stress, including methionine sulfoxide reductase (Liao et al. 2020), thiol oxidoreductase (Lee et al. 2019), and arginine deaminase system (Maybin et al. 2023), were also found increased in the survival bacteria after CAP action.

Interestingly, sometimes CAP-treated bacteria did not exhibit overexpression of antioxidant enzymes but still showed tolerance to CAP, suggesting the existence of other defense mechanisms against oxidative stress in bacteria (Stulic et al. 2019). Protective pigments have the ability to scavenge free radicals and functions as an antioxidant, protecting bacteria against oxidative stress. Staphyloxanthin, is a carotenoid pigment that protects *Staphylococcus aureus* from oxidative stress due to its conjugated double bond (Clauditz et al. 2006). In a study by Liao et al., it was found that the color of *S. aureus* cell granules gradually faded from golden yellow to white in the presence of CAP (2018). And Yang et al. further found that bacteria expressed Staphyloxanthin or its yellow pigment intermediates showed tolerance to CAP. These experiments suggest that the pigments of *S. aureus* have a protective effect against CAP inactivation (2020). In addition to the pigment, Ranjbar et al. found that a CAP can interfere with bacterial glycolytic pathways and affect the rate of bacterial substance metabolic reactions, where the rate of production of the antioxidant succinate and NADPH was increased, suggesting that bacteria can activate defense mechanisms in the presence of CAP (2020). These experiments suggest that bacteria can tolerate the inhibitory effects of CAP through non-enzymatic antioxidant substances.

ROS and RNS are the main factors mediating CAP inhibition, as we have discussed in the previous section. Thus, defense to oxidative stress is crucial in the formation of survival bacteria. In the presence of ROS, RNS, and UV in the CAP, there is bacterial mutation to produce antioxidant-resistant strains. However, the ability of ROS and RNS to induce mutation is average (Patenall et al. 2021) and the amount of UV is low, so the actual formation of mutagenic resistant bacteria is low. The formation of survival bacteria is largely dependent on the alteration of the bacterial phenotype. In the high oxygen concentration environment formed by the CAP, bacteria are forced to alter their energy allocation, allocating a large amount of energy to antioxidant activities, which is reflected in a large upregulation of antioxidant genes in gene expression; while correspondingly less energy is allocated to growth and material metabolism, other energetic physiological activities are suppressed and bacteria survive in a low energy consumption physiological state. The change in the intracellular energy allocation balance reduces the energy for the bacterial metabolism and is considered to result in a viable but non-cultivable (VBNC) state (Liao et al. 2020).

VBNC state is a special physiological state that bacteria enter to adapt to extreme environmental stress (Liu et al. 2021). Bacteria in the VBNC state cannot form a colony in standard medium but can retain their metabolic activity and express toxic proteins (Wu et al. 2020). When bacteria are treated with low-dose CAP, although no colony production could be observed on the medium, the metabolic level of detection suggested that CAP-treated bacteria still had metabolic activity, suggesting that the bacteria were not completely inactivated by the CAP but survived in the VBNC state (Patinglag et al. 2021). The antioxidant system of bacteria is believed to be closely related to CAP-induced VBNC bacteria. Gene expression assays suggest that the antioxidant genes of VBNC bacteria are heavily upregulated and express greater antioxidant capacity than normal bacteria (Liao et al. 2020). Although VBNC bacteria cannot grow on media, they have been found to still remain or even increase the virulence, and thus, the potential pathogenicity of VBNC bacteria should remain a concern (Liao et al. 2021).

The VBNC state of bacteria with low energy consumption and high tolerance to oxidative stress is a self-protective strategy for bacteria to adapt to the extreme environment generated by CAP. At lower doses of CAP, bacteria allocate most of their energy to antioxidant systems that fight against ROS and RNS, and exist in VBNC state, whereas at higher doses of CAP, even though the antioxidant system of the bacteria is heavily upregulated, it is difficult to tolerate the strong oxidative stress and the bacteria are therefore sufficiently inactivated. Considering that VBNC still retain virulence, they still have the potential to infect and cause disease. This suggests that when using CAP bactericidal, the appropriate concentration dose should be chosen to minimize the possibility of bacteria surviving in the VBNC state due to the low concentration of the CAP dose used.

It is worth mentioning that a recent study found that CAP can induce straight response, which leads to the emergence of persisters (Maybin et al. 2023). Persisters and VBNC cells are believed to have the same dormant phenotype, both of which are generated by stress (Kim et al. 2018). It has been discovered that the inactivation of ribosomes via dimerization induced by ppGpp molecule is the likely general mechanism leading to persisters (Song and Wood 2021). Study by Maybin et al. shows that CAP treatment upregulates genes involved in the production of ppGpp, and ppGpp induces upregulation of expression of ribosome modulation factor, thereby altering ribosomal RNA structures, impairing protein synthesis, and upregulating stress-related genes, leading to persister cell formation (2023). This study is the first to report CAP treatment can trigger a stringent response, which is valuable for further study of the mechanism of the production of dormant cells after CAP treatment.

### Heat shock protein activation

In the previous section, we mentioned that CAP can induce the formation of protein–protein cross-links and that this protein aggregation is one of the inhibition mechanisms of CAP. During CAP treatment, denatured and unfolded proteins tend to adhere together, forming insoluble and lethal protein aggregates. Heat shock proteins are a group of chaperone proteins responsible for protecting unfolding proteins and refolding damaged proteins. Vaze et al. found that all heat shock protein deficient mutants were completely inactivated after CAP treatment, in contrast to bacteria with heat shock proteins that partially survived CAP treatment, suggesting that heat shock proteins have the ability to protect bacteria from CAP (2017). In addition, study by Krewing et al. illustrates the mechanism of heat shock protein activation in the presence of CAP (2019b). Under the oxidation of ROS and RNS, the cysteine residues of heat shock proteins are converted to disulfide bonds, which leads to the activation of heat shock proteins. And when heat shock proteins in its active form, it binds unfold proteins to protect them from aggravation. These studies suggest to us that the activation of heat shock proteins may be one of the mechanisms of action of bacterial tolerance to CAP.

### Tolerance mechanisms of biofilms to CAP

Bacterial biofilms are significantly more tolerant to antimicrobial substances compared to planktonic bacteria; correspondingly, biofilms also show greater tolerance than planktonic bacteria in combating CAP treatment (Costello et al. 2021). This is partly because biofilms, like planktonic bacteria, can also adapt to the high oxygen concentration environment generated by upregulating antioxidant stress genes and entering the VBNC state (Mai-Prochnow et al. 2015; Wang et al. 2018), and partly because the structure of biofilms, especially EPS, has an important protective role (Ziuzina et al. 2015). Specifically, the protective effect of the structure of biofilms on the CAP consists mainly of influencing the diffusion of antimicrobial substances and indirectly inducing the formation of persisters.

Dosing of sufficient antibiotic concentrations against the targeted bacteria is hindered in biofilms by the presence of the matrix, which may delay the penetration of the antibiotic molecules into bacterial cells (Ciofu et al. 2022). Similarly, EPS and bacteria located in the outer layer of biofilm have the effect of limiting the diffusion of antibacterial substances. When treating biofilms with CAP, EPS located on the outside of the cell reacts first with the active substance, consuming some of the antimicrobial substance to limit its spread (Limoli et al. 2015; Mai-Prochnow et al. 2021). This tolerance effect is closely related to biofilm biomass, with larger biomass biofilms being less sensitive to the antimicrobial effect of CAP (Gilmore et al. 2018). The principle of the protective effect of EPS is similar to that of the protection of the upper layer of bacteria against the lower layer. Cells located in the top layer of the biofilm are inactivated and can act as a physical barrier to protect cells in the lower layer of the biofilm from CAP penetration (Aboubakr et al. 2020). This protective effect of the upper layer of cells leads to a rapid decline in the number of inactivated bacteria at the beginning of CAP exposure and a slow rate of bacterial decline after the dead cells form a barrier, resulting in a specific biphasic killing curve in terms of bactericidal kinetics (Wang et al. 2018). It is worth mentioning that the protective effect induced by either EPS or the upper cell layer, this protective effect can be considered a “shadowing effect,” similar to the interference effect of organic matter in the medium or wastewater on the CAP (Aboubakr et al. 2020; Bai et al. 2020). This effect is essentially caused by the high reactivity and low penetration of the CAP. Due to high reactivity, CAP tends to react with other organic substances in the environment during transport, which weakens the concentration dose of active substances and makes it more difficult for CAP, which already has limited penetration, to affect bacteria deep in the biofilm. In summary, the interference of the outside of the biofilm with the diffusion of antimicrobial substances protects the bacteria inside the biofilm.

In addition to affecting the diffusion of antimicrobial substances, the presence of EPS also indirectly induces the formation of persisters. EPS has a diffusion-limiting effect on oxygen, nutrients and metabolites—often acidic—around bacteria, allowing these substances to deposition around the bacteria. The substances deposited around bacteria form a low concentration of oxygen or a low acidic environment. Under the action of various environmental stresses, various adaptive response will occur in bacteria, and may induce the formation of persisters under prolonged environmental stimulation (Ciofu et al. 2022; Costello et al. 2021) Arguably, it is the diffusion-limiting effect of EPS that creates a low concentration of oxygen or a low-acid environment around bacteria, allowing biofilm bacteria to be exposed to sublethal stressors in advance, thus pre-empting phenotypic changes and forming persisters. Persisters provide another explanation for the biphasic killing curve. In this opinion, bacteria are killed rapidly in the early stages because persisters have not yet formed, and when persisters are gradually formed under the continuous induction of CAP, there is a reduction in the bacterial inactivation rate in the second half (Flynn et al. 2019).

In addition to these two explanations, dessication of CAP provides the third explanation for the biphasic killing curve. CAP can promote moisture diffusion and improve drying efficiency by etching the surface and affecting the internal microstructure, and has been applied to the food industry as a new drying technology (Du et al. 2022). In the early stage of CAP treatment, water content in the biofilm contributes to the formation of highly reactive species; these include water molecules in biofilms dissociating hydrogen bonds to form hydroxyl radicals under the action of CAP (Kim et al. 2017), and gas phase nitrogen oxide dissolving in water to form nitrate and hydrogen ions, which provides sufficient conditions for further formation of peroxynitrite (Zhou et al. 2018). Therefore, in the early stage of CAP treatment, bacteria receive more highly reactive species, and bacteria are killed quickly. However, in the late stage of CAP treatment, dessication of CAP reduced the water content of the treated surface and the production of highly reactive species, which led to a decrease in the inactivation rate of bacteria. In addition, it has also been suggested that in a relatively dry environment, bacteria will experience cell shrinkage, leading to thickening of the cell membrane, which may hinder the reactive species contained within CAP from moving into the cells (Lee et al. 2015). The dessication of CAP provides an interesting explanation for the biphasic killing curve, but this explanation may not be applicable to the liquid environment, where the effects of plasma dessication may be negligible. In general, the explanation of “shadowing effect” is more widely accepted among the various explanations, but in either case, the presence of the second half of the biphasic killing curve is suggestive of a gradual increase in biofilm tolerance to CAP as it is subjected to CAP treatment.

**Fig. 2:**
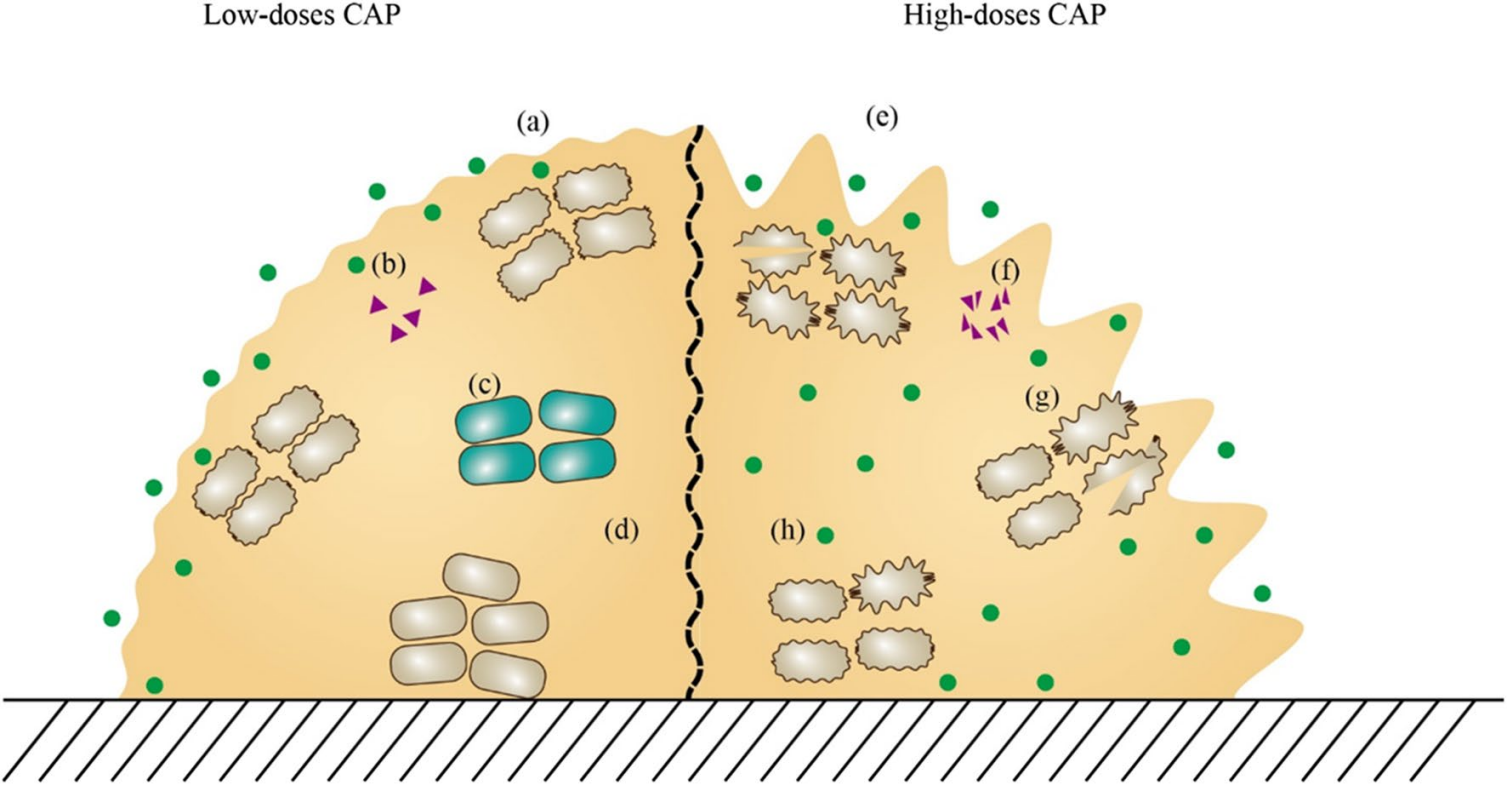
Mechanism of action between CAP and biofilm. At low-doses of CAP, **a** the outer structure of biofilm is slightly damaged, **b** QS signaling molecules are not significantly affected, **c** persisters are induced to form in biofilms, and **d** ROS is restricted in the biofilm surface. At high-dose of CAP, **e** the outer structure of biofilm is seriously damaged, **f** QS signaling molecules are destroyed, **g** the bacterial structure in the biofilm is seriously damaged, and **h** ROS diffuses to the deep layer of biofilm. Green spots represent ROS and RNS in the CAP. Purple triangles represent QS signaling molecules. Gray ellipses represent bacteria in biofilm. Green ellipses represent persister cells

However, the mechanisms by which biofilms tolerate CAP action are not fully understood. As suggested by a study by Kamgang-Youbi et al., biofilm bacteria isolated alone still exhibit greater tolerance to CAP than planktonic bacteria (2008), which seems to be unexplained according to currently known tolerance mechanisms. This suggests that there are still undiscovered mechanisms to be explored for the tolerance of biofilm bacteria to CAP (Fig. 2).

## Bactericidal applications of CAP

### Plasma-activated water (PAW)

CAP-generated reactive particles interact with water molecules to initiate a cascade chemical reaction and form a unique highly reactive mixture known as plasma-activated water (PAW) (Zhou et al. 2018). PAW is considered to be an effective microbicide because it retains the abundant ROS and RNS in the CAP (Li et al. 2017). In addition, the PAW exerts significant shear stress on the treated surface during transport, and the high fluid shear stress is destructive to low-density biofilm aggregates with large spaces (Mai-Prochnow et al. 2021).

The advantage of PAW over direct CAP treatment lies mainly in its greater convenience and safety in use. On the one hand, direct plasma jets have a narrow treatment area and the area of a single inhibition depends entirely on the electrode area, whereas with PAW, it is much easier to disinfect a wide area of treated surface due to the fluidity of the liquid; on the other hand, PAW is an indirect treatment, which means that the treated surface is not affected by electric fields or UV light during use, so potential damage to the sample from direct CAP treatment can be avoided (Yahaya et al. 2021; Zhao et al. 2020).

In addition to convenience and safety in use, the moist environment is suggested to induce more hydroxyl radical generation by promoting the decomposition of water (Huang et al. 2019; Pai et al. 2018). Although PAW is suggested to induce more hydroxyl radical production, PAW is actually less bactericidal than direct CAP treatment. Firstly, short-lived substances, including hydroxyl radicals, are present for a short period of time, so that by the time PAW is used for bactericidal, the activity of these short-lived substances has already disappeared and it is often only the long-lived substances that actually exert a bactericidal effect in PAW (Zhou et al. 2018). Secondly, the concentration of some long-lived ROS in PAW is also significantly reduced, most often by ozone. This is partly due to the quenching effect of water molecules on ozone and partly due to the relatively low Henry’s constant of ozone, meaning it is difficult to enter the liquid phase (Liao et al. 2021; Lis et al. 2018). Finally, PAW does not contain bactericidal factors such as UV, electric field, or discharge particles, as these are present only during the CAP discharge process (Zhou et al. 2018). In summary, the bactericidal effect of PAW is weaker than direct CAP treatment. Nevertheless, data from experiments shows that the bactericidal capacity of PAW still shows significant efficiency (Yahaya et al. 2021; Zhao et al. 2020).

### CAP in combination with antibiotics for antibacterial use

Although CAP exhibits efficient bactericidal action, it has also been suggested in the literature that CAP as an effective antimicrobial agent is able to completely reduce the metabolic activity of bacterial cells or their cultivability, but it is difficult to eradicate them completely (Paldrychová et al. 2020). Also, the low penetrating physical nature of CAP makes it difficult to provide effective killing of bacteria at depth (Wang et al. 2018). This suggests that it is difficult to rely solely on CAP for bactericidal, and therefore, scholars have attempted to use CAP in combination with other antimicrobial agents in an attempt to achieve better bactericidal results.

In recent years, many papers have reported that CAP improves the bactericidal efficacy of antibiotics and even regains the susceptibility of resistant bacteria to antibiotics (Bayliss et al. 2013; Sun et al. 2021). Most scholars have attributed the mechanism by which CAP enhances antibiotic inhibition to the ability of CAP to disrupt the outer cellular layer or the ability of CAP to improve penetration of antibiotics inside the cell, thereby making it easier for antibiotics to enter the cell interior (Brun et al. 2018; Julák et al. 2020; Maybin et al. 2023). However, other mechanisms have also been proposed, such as in a study by Paldrychová et al. where it was suggested that although CAP is not an important inhibitor of QS, it can promote the inhibitory effect of antibiotics on QS signaling molecules (2020). As for the mechanism by which CAP restores susceptibility to resistant bacteria, it has been found that CAP treatment can directly damage resistance genes, which may be the main reason for the restoration of bacterial susceptibility to antibiotics. It is worth noting that although CAP can destroy resistance genes in cells at lower concentration doses, CAP can also disrupt cell membranes, leading to leakage of resistance genes and transmission to other microorganisms through horizontal gene transfer processing, allowing non-antibiotic resistant microorganisms to acquire resistance genes; and at higher doses, the rate of CAP degradation of resistance genes is higher than the rate of leakage, at which point the resistance gene is completely removed (Liao et al. 2018).

However, it has also been suggested in the literature that after CAP pretreatment, bacteria instead show greater resistance to antibiotics. It has been suggested that the development of resistance is associated with a low cellular energy state (Liao et al. 2021). We have discussed previously that VBNC bacteria are in a state of low metabolic activity. Cellular energy metabolism is inhibited in the hypometabolic state, which allows energy-dependent antibiotic targeting pathways, such as DNA replication, protein synthesis, and folate biosynthesis, to be less active, which reduces the efficiency of binding between the antibiotic and its target (Conlon et al. 2016). In addition, scholars have generated discussions on whether antibiotics trigger the formation of endogenous ROS and generate oxidative stress leading to bacterial death (Kohanski et al. 2007; Van Acker and Coenye 2017). If antibiotics rely on ROS to mediate bactericidal, as the bactericidal effect of CAP also has a predominantly oxidative stress, the antioxidant response of bacteria which is upregulated by the pre-treatment of CAP will also lead to resistance to antibiotic-mediated oxidative stress.

The two sides of the CAP-antibiotic combination are the following: the possible induction of mutations and adaptive changes in bacteria when pretreated at sub-lethal doses of CAP, leading to the development of resistance to antibiotics; and the slowing down of the inactivation rate of resistance genes, leading to horizontal gene transmission across the cell membrane. In contrast, at the right dose of CAP, CAP is able to somewhat disrupt the outer layer of the cell, facilitating the entry of antibiotics into the cell interior; and promoting effective inactivation of resistance genes. This suggests that CAP should be used in combination with antibiotics at the appropriate dose.

### CAP in combination with advanced oxidation processes for antibacterial use

So-called advanced oxidation processes are processes or reactions capable of producing highly reactive radical intermediates—particularly hydroxyl radicals. Hydroxyl radicals are the neutral form of the hydroxide ion and have the strongest redox potential of all oxidation-active substances. There are many ways to generate hydroxyl radicals, involving various chemical, photochemical, biochemical, or electrochemical reactions, and many process technologies, including CAP, are advanced oxidation processes. In a review by Fan and Song, advanced oxidation processes are systematically described and it is suggested that the combined application between advanced oxidation processes can generate more hydroxyl radicals than when applied alone (2020).

Ultrasound is a common advanced oxidation process that is suggested for surface disinfection of fresh produce at low frequencies and is often used in combination with other physical methods, chemical reagents, and natural compounds. The application of ultrasound to a liquid interface induces the formation of cavitation bubbles, which collapse violently as they increase in size until they reach a critical size, with the collapsed bubbles generating instantaneous extreme heat and shear. The high transient temperatures and pressures lead to the thermal fragmentation of water molecules, resulting in various ROS that induce cell death (Wang and Wu 2022). The mechanism for the combined application of ultrasound and CAP is considered to be primarily that the high temperature and pressure generated by ultrasound causes damage to the cell membrane, allowing the CAP to enter the cell interior more easily (Charoux et al. 2020).

Hydrogen peroxide has a wide range of bactericidal and inhibitory activities, and upon contact with ferrous ions in the mixture, it can undergo a Fenton reaction to form hydroxyl radicals (Song and Fan 2020). However, the antimicrobial efficiency of hydrogen peroxide as a disinfectant is usually low, so scholars have tried to apply hydrogen peroxide in combination with other on technologies. It was found that the combined use of CAP and hydrogen peroxide has a synergistic effect. CAP produces small amounts of ozone, which in turn reacts with hydrogen peroxide to produce hydroxyl radicals (Jiang et al. 2017; Song and Fan 2020). In addition, hydroxyl radicals are known to be highly reactive and extremely unstable, and can only exist transiently in the environment. However, hydrogen peroxide microdroplets are believed to become charged when it passes through the CAP field, increasing the stability of the nano-sized droplets. This is considered to be another reason for the high efficiency of the CAP when coupled with hydrogen peroxide (Pyrgiotakis et al. 2015).

### CAP in combination with chemical disinfectants for antibacterial use

Chemical disinfectants are widely used in various fields. However, various disadvantages of chemical disinfectants have gradually made them inadequate for disinfection needs. Firstly, the bactericidal efficacy of chemical disinfectants is inadequate, especially when disturbed by organic or inorganic materials in the environment (Chaplot et al. 2019; Fan and Song 2020). Secondly, the penetration capacity of chemical disinfectants is limited, making it difficult to reach bacteria located in protected locations such as cracks, stomata, and cut voids (Fan and Song 2020; Song and Fan 2020). Finally, some chemical disinfectants, represented by chlorine, have been shown to be potentially carcinogenic or mutagenic, raising concerns about the effects on human health and safety (Chaplot et al. 2019; Jiang et al. 2017).

As a result, the use of chemical disinfectants in combination with other germicidal technologies has been sought to improve the disadvantages of chemical disinfectants in terms of insufficient germicidal efficacy at low doses and excessive side effects at high doses. CAP has been shown to have a synergistic effect on chemical disinfectants, improving their germicidal efficacy. It is generally accepted that the combined bactericidal mechanism of CAP and chemical disinfectants is due to the disruption of cell membranes by CAP, which facilitates the entry of chemical disinfectants into the cell, thereby enhancing bactericidal efficacy (Chaplot et al. 2019; Yadav and Roopesh 2022). However, it has also been suggested in the literature that CAP can act directly to activate chemical disinfectants and promote the formation of more ROS (Qian et al. 2020).

## Summary

This paper systematically reviews the mechanisms of CAP inhibition and bacterial tolerance to CAP. It is clear that CAP inhibition and tolerance of survival bacteria are a set of closely related mechanisms: CAP relies mainly on ROS and RNS for inhibition, while survival bacteria tolerate mainly through antioxidant systems; CAP induces protein-protein cross-link formation, while survival bacteria tolerate protein aggregation through heat shock proteins; CAP degrades biofilms by disrupting EPS, while survival bacteria rely on EPS to tolerate CAP. The comparison of inhibition and tolerance has led to a more comprehensive understanding of the mechanism of action of CAP, which has also contributed to our thinking about the relationship between other antimicrobial substances and bacteria.

Interestingly, there are more known mechanisms of CAP inhibition than tolerance for survival bacteria. It may be possible to find a counterpart to the extra inhibition mechanism in the tolerance mechanism of the survival bacteria, e.g., by modulating the QS to tolerate the action of the CAP. This review is helpful for the exploration of undiscovered tolerance mechanism of the survival bacteria, and a clearer understanding of the mechanism will help to improve the bactericidal application of CAP and even other advance oxidation process more effectively. In conclusion, this review shows that CAP is a highly effective bactericidal and it is difficult for bacteria to survive at appropriate dose of CAP.
